# The Proposed Amalgamation of St. George's and Westminster Hospitals

**Published:** 1913-07-12

**Authors:** 


### THE PROPOSED AMALGAMATION OF ST. GEORGE'S AND WESTMINSTER HOSPITALS.

### ST. GEORGE'S HOSPITAL.

Notice has been given that at the Special Court of the Governors of St. George's Hospital summoned to meet on Thursday, 10th inst., the following resolutions will be proposed:

That an amalgamation of St. George's Hospital with the Westminster Hospital is desirable.

That it is desirable to effect a sale of the site of St. George's Hospital and of the adjoining houses, Nos. 1, 3, 5, 7, and 9 Knightsbridge, together with the buildings thereon, and that the House Committee be authorised to enter into a contract for sale, on the basis of an offer from a prospective purchaser, which will be produced to the Court, and upon such terms as the House Committee may be advised, and to pay such commission for the introduction of the purchaser as they may think fit.

That the House Committee be authorised to make such arrangements as they may think desirable with the Duke of Westminster as regards that portion of the hospital site purchased from the Westminster Estate and conveyed by the deed of October 31, 1906, and to pay such sum or sums as they may be advised for obtaining the release of any rights reserved to the Duke by such deed.

That it be a term of the amalgamation that the governors of the two hospitals shall become governors of the amalgamated institution, and that the consultant and honorary staff of the two hospitals shall retain their positions in the amalgamated institution, vacancies as they occur being filled by the new governing body.

That a Joint Committee composed of six members of the House Committee of each hospital (or such other number as may be mutually agreed) shall be appointed by the respective House Committees to select a site or sites for and obtain the erection of the amalgamated hospital, with all necessary and proper adjuncts, and that the House Committee be authorised to enter into a contract or contracts for the purchase of such site or sites, and for the erection thereon of the new hospital, with its adjuncts, on such terms as they may be advised, and at such cost as they shall consider reasonable, the new hospital to provide not less than 500 beds.

That the House Committee be empowered to apply for any Act of Parliament and to enter into any contracts and take any other steps which may be necessary or desirable for giving effect to the foregoing proposals, and to cause the corporate seal of the hospital to be affixed to any documents necessary for the foregoing purposes.

As the meeting will be held after we go to press this week, a full report of the proceedings will appear in The Hospital for the 19th inst.

### THE WESTMINSTER HOSPITAL.

A Bill which is now before Parliament and which has passed the House of Commons, entitled The Westminster Hospital Act, 1913, attained its second reading in the House of Lords on the 8th inst., and was referred to a Select Committee to be appointed. It is an Act to empower the President, Vice-Presidents, Treasurers, and Governors of the Westminster Hospital to acquire lands for and to erect a new hospital. It is further to authorise the sale and disposal of the site of Westminster Hospital. It contains provisions as to the Nurses' Home and amendment of Order of 1904, and also as to its Medical School, and the Incurables Fund. It will thus be seen that the proposed amalgamation and arrangements are proceeding in due order to put each institution in the position to complete it and to combine for the purpose of erecting a new hospital of 500 beds.

